# Data-based Decision Rules to Personalize Depression Follow-up

**DOI:** 10.1038/s41598-018-23326-1

**Published:** 2018-03-22

**Authors:** Ying Lin, Shuai Huang, Gregory E. Simon, Shan Liu

**Affiliations:** 1 Department of Industrial Engineering, University of Houston, 4722 Calhoun Road, Houston, TX 77204 United States; 2 Department of Industrial and Systems Engineering, University of Washington, Box 352650, Seattle, WA 98195 United States; 3 Kaiser Permanente Washington Health Research Institute, 1730 Minor Ave, Suite 1600, Seattle, WA 98101 United States; 4 Psychiatry and Behavioral Sciences, University of Washington, Box 356560, Seattle, WA 98195 United States

## Abstract

Depression is a common mental illness with complex and heterogeneous progression dynamics. Risk grouping of depression treatment population based on their longitudinal patterns has the potential to enable cost-effective monitoring policy design. This paper establishes a rule-based method to identify a set of risk predictive patterns from person-level longitudinal disease measurements by integrating the data transformation, rule discovery and rule evaluation. We further extend the identified rules to create rule-based monitoring strategies to adaptively monitor individuals with different disease severities. We applied the rule-based method on an electronic health record (EHR) dataset of depression treatment population containing person-level longitudinal Patient Health Questionnaire (PHQ)-9 scores for assessing depression severity. 12 risk predictive rules are identified, and the rule-based prognostic model based on identified rules enables more accurate prediction of disease severity than other prognostic models including RuleFit, logistic regression and Support Vector Machine. Two rule-based monitoring strategies outperform the latest PHQ-9 based monitoring strategy by providing higher sensitivity and specificity. The rule-based method can lead to a better understanding of disease dynamics, achieving more accurate prognostics of disease progressions, personalizing follow-up intervals, and designing cost-effective monitoring of patients in clinical practice.

## Introduction

The extended use of Electronic Health Record (EHR) provides an abundance of clinical measurements that may help to predict patients’ disease progressions. Leveraging this rich information can accelerate the transition from one-size-fits-all monitoring guidelines to personalized monitoring strategies. For example, approximately 30 million Americans are using antidepressant medication for depression treatment with the goal of achieving complete remission and preventing relapse of depression^1,2^. Due to potential side effects of the medication, the U.S. Food and Drug Administration (FDA) has made recommendations to closely monitor patients taking antidepressants^3,4^, with the most stringent recommendation being 7 visits in 12 weeks for children and adolescent and one visit every 6 months or annually for adults^5^. Due to the lack of evidence from empirically designed monitoring strategy, studies have consistently documented far less monitoring in practice than the FDA recommendations, e.g. one study noted that only 23% of patients have received the FDA-recommended level of care at 12 weeks^6^. Furthermore, existing recommendations regarding follow-up intervals do not consider significant heterogeneity in depression treatment outcomes. Accurate prognostics of patients’ depression severities can provide evidence for identifying which patient should be closely monitored in clinical practice, holding promises for more efficient and cost-effective monitoring strategies.

Statistical analysis of the EHR data has the potential to identify risk predictive factors for disease progression and provide accurate prognostics for patients’ health outcomes. Recent advances in machine learning have provided a variety of prognostic models, such as logistic regression, support vector machine (SVM), and random forest. However, these prognostic models for predicting individuals’ disease progressions from EHR data are inadequately implemented in practice due to the following challenges. First, machine learning models such as SVM and random forest deal with the complex interactions between predictive factors, but they lack interpretability to understand disease etiology and support medical decision making^7^. In addition, since disease progression is a complex and dynamic process, understanding its etiology requires repeated clinical measurements over time rather than relying only upon a baseline profile^8^. Identifying the predictive factors for disease progression from time-varying and irregular clinical measurements poses a significant challenge. Furthermore, the widely reported heterogeneity on disease progression further increases this challenge. For instance, five broad trajectory patterns have been found in a depression treatment population from a U.S. EHR dataset^9^. Rather than examining subpopulations with distinct depression trajectory patterns, existing research on depression prognostics focuses on identifying risk factors that are associated with the outcome of interest, which are essentially global associations on the population level^10–12^. Logistic regression^10^, structural equation modeling^11^ and regression analysis^12^ are commonly used methods to model the association between depression progression and risk factors. Consequently, risk factors identified from these models only reflect the average effects over a population and are inadequate to be used for decision making on the individual patient level^13^.

The aim of this study is to establish a rule-based analytic framework to identify a set of risk predictive longitudinal patterns from the EHR data to personalize depression prognostics and adaptive monitoring. Rule-based analysis is particularly useful for identifying a set of risk patterns that segment the population into subgroups, with individuals in the same group share similar patterns and thus tend to develop similar outcomes^14^. A rule describes the range of values on one or more risk factors that either indicates increased or decreased risk for disease progression. Rules generated on time-varying risk factors provide some natural semantics to define the risk predictive longitudinal patterns; since a rule may indicate a low-risk signal or warning signal for monitoring. By identifying the unknown rules from observations, a set of risk predictive rules can be considered as a set of medical signals, providing us with a risk estimation by looking into the risk patterns endorsed by each individual^14^. Next, by integrating the risk predictive rules with monitoring frequencies, a set of rule-based monitoring strategies can be developed to enable frequent monitoring of individuals with warning signals, and to allow less frequent monitoring of individuals with low-risk signals.

**Figure 1:**
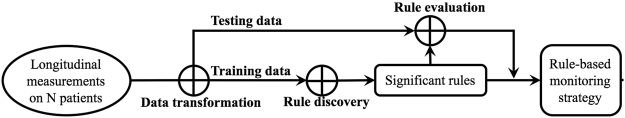
The rule-based analytic framework for longitudinal pattern discovery and adaptive monitoring.

Specifically, our method integrates data transformation, rule discovery, and rule evaluation by following the steps in Fig. 1. We first transform the EHR data of each individual to his/her disease severity assessment and measurements of a set of risk predictors. Then, we randomly split the data into training and testing data. We discover a set of rules on the training population, and further investigate the risk levels of subgroups endorsing/un-endorsing these rules on both training and testing populations. Association between the identified risk predictive rules and individual disease severities is further studied on the testing population. We then extend the identified rules to create rule-based monitoring strategies and adaptively monitor the testing population.

## Methods

### Data description

The Mental Health Research Network (MHRN) data are drawn from the EHR of four health systems participating in the MHRN (HealthPartners, and the Colorado, Washington, and Southern California regions of Kaiser Permanente)^15^. The data include Patient Health Questionnaire (PHQ)-9^16^ results between years 2007 and 2012. The data also include relative time between PHQ-9 measures, treatment status, type of providers (primary care, specialist, mental health), individuals’ age, sex, and the Charlson comorbidity score (a standard indicator of medical disease burden). These variables are likely to be available in most electronic health record or depression outcomes monitoring system.

We focus our study on 1,762 individuals receiving ongoing treatment (defined as either psychotherapy visit in the prior 90 days or filled prescription for antidepressant in the prior 180 days). To capture the depression progression process, we focus on the individuals who are frequently monitored with no less than 3 measurements in the first 6 months and at least one measurement in the next half year. We randomly separate 1,200 individuals (68% data) into a training set to build the prognostic model and identify a set of predictive rules. The training set is selected using the simple random sampling method^17^, which has been widely used to avoid sampling bias. Then we validate the prediction capability and association with underlying disease severity of the identified rules on the remaining individuals (testing data).

The study only included de-identified patients’ electronic health records and all analyses have been carried out in accordance with the approved guidelines by institutional review board through Kaiser Permanente Washington Health Research Institute (KPWRI) and MHRN. The data that support the findings of this study are available from the MHRN, but restrictions apply to the availability of these data. Data are however available from the authors upon reasonable request and with permission of MHRN.

### Data transformation

We build a prognostic model based on measurements within 6 months to predict an individual’s depression severity in the following 6 months. For each individual, we transform the measurements in the first 6 months (referred as the predicting period) and the following 6 months (referred as the responding period) to predictive factors and depression severity, respectively. In total, we generate 45 factors (shown in Supplementary eTable 3) that mainly come from three categories, i.e., statistical summarizations, progression trajectories, and non-random longitudinal patterns. The depression severity of each patient can be assessed using the average PHQ-9 score in his/her responding period. Based on the conventional classification of PHQ-9 scores, patients with an average PHQ-9 score lower than 10 are in the low-risk group and otherwise can be regarded as in the depressive group^16^. We define the outcome, *Y*_*i*_, as 1 if the patient *i*, *i* = 1, …, *N*, is in low-risk group in the following 6 months and 0 otherwise.

### Statistical summarizations

For each individual, we summarize the longitudinal PHQ-9 scores, Charlson comorbidity scores and the 9^th^ question scores on suicide ideation in terms of their statistical measurements, which include first value, maximal value, minimal value, range, median value, 25% and 75% percentile values, average value, and volatility (i.e. standard deviation). The PHQ-9 scores can be further segmented into five depression severity levels using the conventional classification, including minimum (0–4), mild (5–9), moderate (10–14), moderately severe (15–19) and severe (20–27)^15^. We also summarize the percentage of PHQ-9 scores in each depression level.

### Progression trajectories

As demonstrated in Lin *et al*.^9^, fluctuation of PHQ-9 scores can be predictive for depression progression. To extract these temporal patterns, we consider the deepest decrease and deepest increase between two consecutive PHQ-9 scores together with the volatility of the difference between nearby PHQ-9 scores. We further use the time stamp of the first observation, number of observations, observing density and the latest PHQ-9 score, to describe the irregularity and sparsity of the individual’s EHR data. The observing density is defined as the number of observations divided by the time span of all observations.

### Non-random longitudinal patterns

In addition to the factors mentioned above, we adopt control theory to further extract the non-random longitudinal patterns in individuals’ PHQ-9 scores. The moving range control charts and the control rules including the Western Electronic rules (WE) and Nelson rules^18^ (NC) are used to capture the non-random patterns in the depression progression (details see eAppendix 1).

### Rule discovery

We assume individuals can be categorized into risk groups by a set of longitudinal patterns. Each pattern is characterized as a rule over factors (as described in data transformation) extracted from time stamped measurements. For example, individuals endorsing a rule consisting of the latest PHQ-9 score greater than 17 and the volatility of PHQ-9 score lower than 7 are predicted to have higher probability of depression in the next 6 months. We use the RuleFit^19^ to discover these longitudinal patterns (represented as rules) predictive of depression severity. RuleFit is a high-dimensional computational algorithm for rule discovery from a large number of candidate risk factors. It generates the rules by first exhaustively searching for candidate rules over the potential risk factors in the “rule generation” phase and pruning out the redundant and irrelevant rules in the “rule pruning” phase^19^ (details see eAppendix 2).

A set of predictive rules is discovered where each individual rule can segment the population into subgroups with distinct disease severities. We calculate the proportion of low-risk patients (*Y*_*i*_ = 1) in each rule endorsing group. Then, we select the rules that have high prevalence of either depressive patients or low-risk patients in their endorsing groups. In other words, the rules that lead to endorsing groups which have an equal mix of depressive and low-risk patients are not predictive and thereby discarded. We then name the rules as either increasing or decreasing risk rules. Specifically, the decreasing risk rules which have high proportion of low-risk patients in their endorsing groups indicate that patients satisfying these rules are less likely to have depression in the next 6 months. The increasing risk rules, on the other hand, have low proportion of low-risk patients in their endorsing groups and indicate higher risk of depression if these rules are endorsed.

### Rule evaluation

Rules identified from the previous step are predictive of depression severity on the training population. To evaluate the statistical significance of these rules on the testing population, we first test whether the depression severities in the rule endorsing and un-endorsing groups are significantly different. We apply the Mann-Whitney-Wilcoxon test on each rule, which is a non-parametrical statistical test for deciding whether two independent samples come from the same distribution. We further build a rule-based prognostic model to predict individuals’ disease severities based on the rule endorsements. The rule-based prognostic model uses the item response theory (IRT)^14,20^ to model the relationship between individuals’ endorsements on each rule and their disease severities. It assigns each individual a latent variable denoting the underlying disease severity, and models the likelihood of endorsement of each rule as a function of the individual’s disease severity (details see eAppendix 2). The latent variable learned from IRT is normalized to the interval between 0 and 1 to represent the disease severity of each individual.

### Rule-based adaptive monitoring

The endorsement of each risk predictive rule provides evidence for adaptive monitoring in the following 6 months by stratifying the individual’s depression severity into risk levels. Specifically, individuals endorsing the decreasing risk rules are less likely to have depression in the following period and may be less frequently monitored; while individuals satisfying the increasing risk rules should be closely monitored. Therefore, each rule can be extended to a rule-based monitoring strategy by applying both individual-rule based policies and multiple-rules based policies outlined in Table 1. In the individual-rule based monitoring, for each decreasing risk rule, individuals will be monitored if the rule is not endorsed and not monitored otherwise; for each increasing risk rule, individuals will be monitored if the rule is endorsed and not monitored otherwise. In the multiple-rules based monitoring, we segment the population into four groups based on the endorsements of increasing and decreasing risk rules, including un-endorsing any of increasing risk rules and any of decreasing risk rules (Group 1), endorsing any of increasing risk rules but un-endorsing all decreasing risk rules (Group 2), un-endorsing all increasing risk rules but endorsing any of decreasing risk rules (Group 3), and endorsing all rules (Group 4). Individuals in Group 2 tend to have higher disease risk and the multiple-rules based monitoring suggests to closely monitor them. The individuals in Group 3 is more likely to be healthy patients so multiple-rules based monitoring suggests to not monitor them. The Group 1 and Group 4 are uncertain groups. By considering different monitoring decisions in these uncertain groups, we compare four scenarios in the multiple-rules based monitoring, i.e. monitoring both groups, not monitoring both groups, monitoring the group of endorsing both increasing and decreasing risk rules and monitoring the group of un-endorsing all rules.

**Table 1:** (**a**) Individual-rule based monitoring strategies in the next 6 months. (**b**) Multiple-rules based monitoring strategies in the next 6 months.

**(a)**						
**Rule type**			**Rule endorsement**			
			**Endorsed**		**Unendorsed**	
**Increasing risk rule**			Monitor		Not monitor	
**Decreasing risk rule**			Not monitor		Monitor	
**(b)**						
	**Increasing risk rule**	**Decreasing risk rule**	**Scenario 1**	**Scenario 2**	**Scenario 3**	**Scenario 4**
Group 1 (2.3%)	unendorsed	unendorsed	Monitor	Not monitor	Not monitor	Monitor
Group 2 (45.6%)	endorsed	unendorsed	Monitor	Monitor	Monitor	Monitor
Group 3 (41.8%)	unendorsed	endorsed	Not monitor	Not monitor	Not monitor	Not monitor
Group 4 (10.3%)	endorsed	endorsed	Monitor	Not monitor	Monitor	Not monitor

Note: the population is segmented into four groups including un-endorsing any of increasing risk rules and any of decreasing risk rules (Group 1), endorsing any of increasing risk rules but un-endorsing all decreasing risk rules (Group 2), un-endorsing all increasing risk rules but endorsing any of decreasing risk rules (Group 3), and endorsing all rules (Group 4) (percentage of patients in each group is presented in brackets). Four monitoring scenarios are considered: monitoring groups 1, 2 and 4 (Scenario 1); monitoring group 2 (Scenario 2); monitoring groups 2 and 4 (Scenario 3); monitoring groups 1 and 2 (Scenario 4).

To evaluate the efficiency of rule-based monitoring, we compare several monitoring strategies by estimating the number of depressive patients (PHQ-9≥10) in the next 6 months that are correctly monitored (true positives). We assume under the status quo, all patients are monitored every 6 months, which may lead to unnecessary monitoring of low-risk patients. We also consider a PHQ-9 based strategy, which monitors the patient if his/her last-period PHQ-9 score is 10 or greater. Under rule-based monitoring, we consider both using individual rules and combining all top predictive rules.

## Results

### Rule discovery

**Figure 2:**
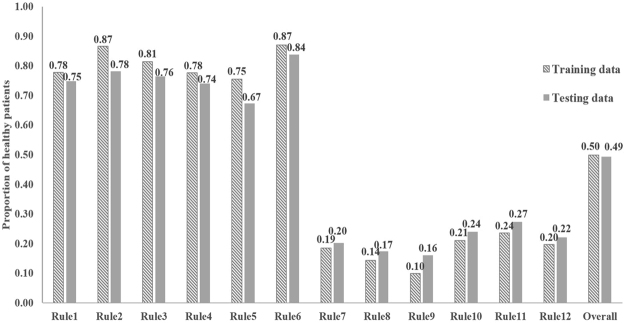
Proportion of low-risk patients (average PHQ-9 score <10) in rule-endorsing group.

We apply the RuleFit model on the training data and identify 46 rules. By calculating the proportion of low-risk patients in each rule endorsing group, we select 12 top predictive rules listed in Table 2, whose proportion of low-risk patients is lower than 30% or greater than 70%. The proportions of low-risk patients in rule endorsing groups as well as the overall proportions of low-risk patients in the training and testing populations are summarized in Fig. 2. The identified rules are distinguished into 6 decreasing risk rules and 6 increasing risk rules. For example, in the first decreasing risk rule (Rule 1), if the observations in a 6-months window of an individual have deepest increase between consecutive PHQ-9 scores smaller than 7.50 and 75 percentiles of PHQ-9 scores smaller than 14.62, the individual is less likely to be high risk in the next 6 months. It can be observed that age, sex and the latest PHQ-9 score are important risk factors in the identified rules, which are consistent with the significant risk factors identified from the logistic model, as shown in eAppendix 3 (Supplementary eTable 2). However, rule-based method may be more powerful in capturing the interactions between significant risk factors and their critical ranges that improve the predictability and interpretability than the logistic regression model. For example, it finds that (1) males with a less severe depression trajectory (mean score on the 9th question lower than 0.71 and fewer than 38% observations being in moderately severe depression (15 ≤ PHQ-9 < 20)) are less likely to have depression in the future, and (2) patients in young and middle adulthoods (age <65) having more than 23% severe depression measurements on their PHQ-9 records are more likely to be depressed in the future. In addition, the identified rules include rich information for describing the depression trajectories. For example, the rule of latest PHQ-9 score greater than 17 and volatility of PHQ-9 score smaller than 7 indicates stable high PHQ-9 scores in the depression trajectory and the rule of latest PHQ9 score lower than 8.50 and maximal PHQ9 score lower than 16.50 indicates stable low depression severity.

**Table 2:** 12 top rules identified by the RuleFit model.

	Decreasing risk rules		Increasing risk rules
Rule 1	Deepest increase between consecutive PHQ9 scores <7.50 & 75 percentile of PHQ9 score <14.62	Rule 7	Observing density >0.03 & Minimal PHQ9 score >8.50
Rule 2	25 percentile of PHQ9 score <6.13 & Volatility of PHQ9 score <9.64	Rule 8	Minimal PHQ9 score >9.50 & Volatility of difference between nearby PHQ9 scores <4.75
Rule 3	75 percentile of PHQ9 score <15.88 & Percentage of moderate depression <0.39	Rule 9	Latest PHQ9 score >17.50 & Volatility of PHQ9 score <7.33
Rule 4	Deepest decrease between consecutive PHQ9 scores >2.50 & 75 percentile of PHQ9 score <14.12	Rule 10	Minimal PHQ9 score >6.50 & 75 percentile of PHQ9 score >14.88
Rule 5	Sex is male & Mean of 9^th^ question scores <0.71 & Percentage of moderately severe <0.38	Rule 11	Age <65 & Percentage of severe depression >0.23
Rule 6	Latest PHQ9 score <8.50 & Maximal PHQ9 score <16.50	Rule 12	Deepest decrease between consecutive PHQ9 scores <13.50 & Mean of PHQ9 scores >14.73

Note: the cut-off values of the variables in the rules were automatically determined by RuleFit for maximum statistical prediction power.

### Rule evaluation

**Figure 3:**
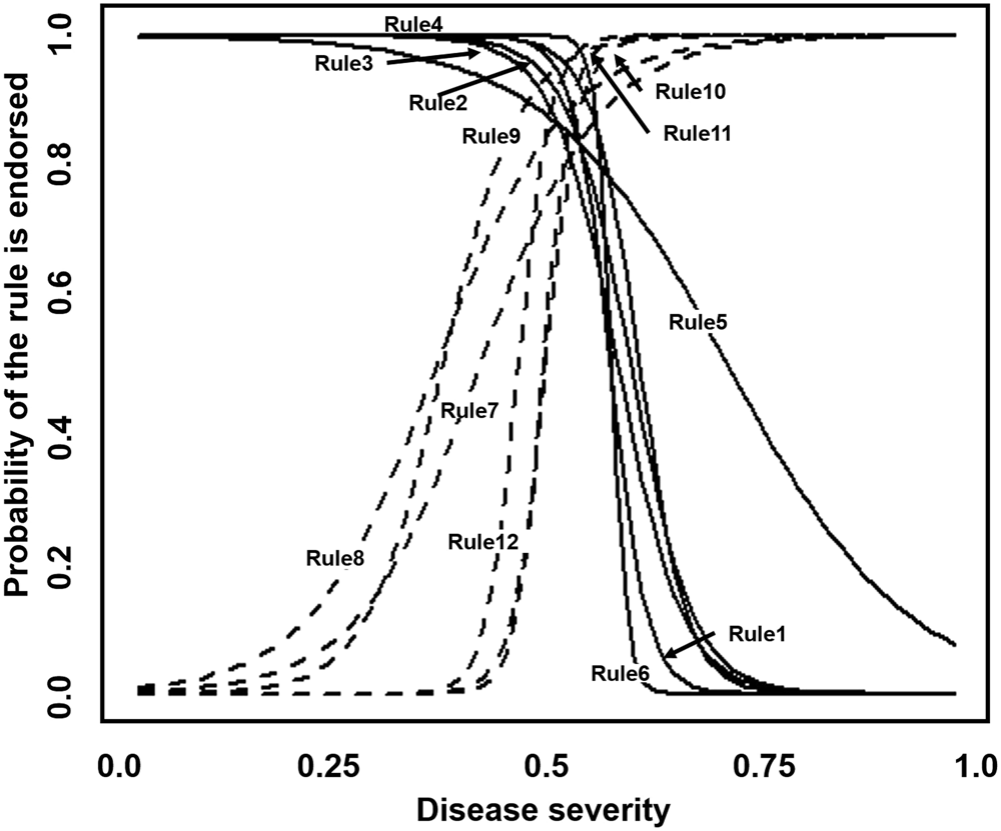
The associations between rule endorsements and depression severity of 12 rules. Each curve represents the probabilities of endorsing a rule under various depression severity. The increasing risk rules are plotted in dash lines and the decreasing risk rules are plotted in solid lines.

The disease severity of each patient is normalized between 0 and 1 with larger value indicating more severe depression. We evaluate the prediction capability of an individual rule by examining the distributions of depression severity in the rule endorsing and un-endorsing groups in Supplementary eFigure 2. The distributions are plotted for a randomly selected decreasing (increasing) risk rule. It can be observed that most patients who endorse the decreasing risk rule tend to have lower depression severity than the patients who do not endorse the rule, and vice versa for the increasing risk rule. P-values of the test statistics on all rules are lower than the significant level (0.01), which indicate each rule segments the population into subgroups with significantly different depression severities. We further investigate the association between the rules and depression severities by using the item characteristic curve (ICC) defined in eAppendix 2. The probability of endorsing each rule based on the estimated disease severity is calculated. The associations between rule endorsements and disease severities of 12 rules are plotted in Fig. 3; the decreasing rules are drawn by solid lines and increasing risk rules are drawn by dash lines. It can be observed that the decreasing risk rules are more likely to be satisfied than the increasing risk rules when the individual has low severity of depression; while the endorsement of increasing risk rules is more likely to be seen when the individual is severely depressed.

Next, we estimate the disease severity of each individual by applying the rule-based prognostic model (described in Methods rule evaluation section) on the identified rules. To evaluate the goodness of prediction, we compare the prediction accuracy of the rule-based prognostic model using 12 identified rules with the RuleFit, logistic regression, and SVM models trained on all 45 factors or the factors included in the identified rules. The prediction accuracies of these models are evaluated using the average area under the curve (AUCs) on the testing population. The results are summarized in Table 3. The rule-based prognostic model outperforms the other methods, which demonstrates the prediction capability of the 12 rules. We also note that the subset of factors included in the 12 rules is predictive of health outcome. The distributions of disease severities estimated from the rule-based prognostic model in the depressive and low-risk groups are compared using the boxplot in Supplementary eFigure 3, where the depressive group has higher predicted disease severities than the low-risk group. Overall, endorsement of the increasing and decreasing risk rules are predictive of the individuals’ depression severity.

**Table 3:** Prediction accuracy of several methods on testing data.

Model	Rule-based prognostic model	RuleFit		Logistic regression		SVM	
		All factors	Significant factors	All factors	Significant factors	All factors	Significant factors
**AUC**	0.83	0.82	0.82	0.81	0.81	0.81	0.81

### Rule-based adaptive monitoring

**Figure 4:**
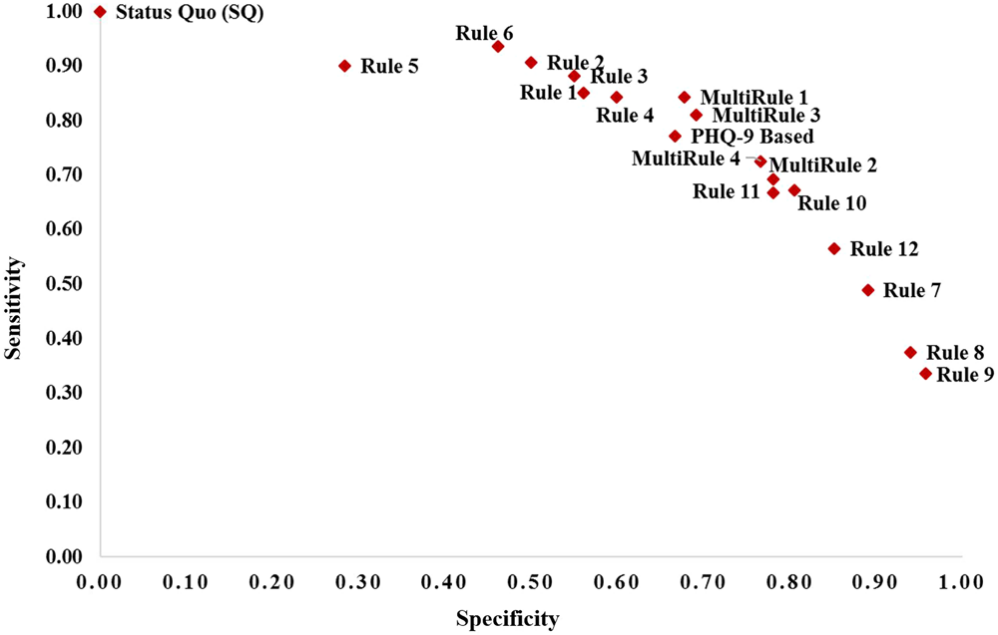
Comparison of sensitivity and specificity in all monitoring strategies.

The monitoring outcomes of various strategies using testing data are shown in Fig. 4 and Table 4. It can be observed that the multiple-rules based monitoring strategies in scenario 1 (MultiRule 1) and scenario 3 (MultiRule 3) outperform the latest PHQ-9 score based strategy (PHQ-9 Based) by providing higher sensitivity and specificity.

**Table 4:** Monitoring outcomes of all strategies (N = 562 patients).

Strategy	Sensitivity	Specificity
Rule 9	0.34	0.96
Rule 8	0.38	0.94
Rule 7	0.49	0.89
Rule 12	0.56	0.85
Rule 10	0.67	0.80
Rule 11	0.67	0.78
MultiRule 2	0.69	0.78
MultiRule 4	0.73	0.77
PHQ-9 Based	0.77	0.67
MultiRule 3	0.81	0.69
MultiRule 1	0.84	0.68
Rule 4	0.84	0.60
Rule 1	0.85	0.56
Rule 3	0.88	0.55
Rule 2	0.91	0.50
Rule 6	0.94	0.46
Rule 5	0.90	0.28
Status Quo (SQ)	1.00	0.00

## Discussion

We establish a general rule-based analytic framework to identify the longitudinal patterns for predicting disease severity in a heterogeneous patient population, and provide actionable knowledge to support the design of adaptive monitoring strategy. We apply this framework on a depression treatment population and demonstrate that the rule-based method can efficiently identify risk predictive longitudinal patterns from sparse and irregular measurements of depression severity (i.e. PHQ-9 score), demographic factors (age and sex), and Charlson comorbidity scores within a 6-months monitoring period. 12 longitudinal patterns are found to be predictive of depression severity in the following 6 months.

The 12 identified rules include time-varying measurements such as PHQ-9 scores as well as time invariant measurements such as age and sex. These risk factors have been identified as significant predictors for depression progression in the literature^15,21–23^, which provide validation for our rule-based analysis. However, existing studies only reflect the average effects of risk factors over the whole population and ignore their interactions. Rule-based analysis may be superior at capturing complex interactions between risk factors and further characterizing depression progression in each risk group. For instance, a two-year follow-up study on 352 patients responding to treatment of major depression (MD) found that females were more likely than males to experience a MD in any month of the study, and marginally more likely to experience a relapse^24^. In addition, depression trajectories characterized by historical measurements of the PHQ-9 questionnaire have been found predictive of depression progression^25,26^. However, studies considering the interactions between sex and depression trajectories are limited. Costello *et al*. used semi-parametric group-based modeling to explore risk factors associated with trajectories of depressed mood from adolescence to early adulthood, and found females were more likely to be classified into stable-low depressed mood, early-high depressed mood and late-escalating depressed mood patterns versus no depressed mood^27^. The associations discovered from this retrospective data analysis are inadequate to predict future depression progression. Complementary to evidence from the literature, our study finds males with a lower than 0.71 mean score on the 9^th^ question and fewer than 38% observations being in moderately severe depression (15 ≤ PHQ-9 < 20) are less likely to have depression, which demonstrates sex has an impact on the depression trajectory. Furthermore, several studies examined depression onset in different age groups^21^. However, there is a lack of agreement on the relationship between age and depression onset. A study on the trajectory of depression symptoms across 2,320 adults’ life span has found that depressive symptoms were highest in young adulthood, decreased across middle adulthood, and increased again in older adulthood^28^. In our study, we find patients in young and middle adulthoods (age <65) having more than 23% severe depression measurements on their PHQ-9 records are more likely to be depressed in the future.

By evaluating the prediction capability of individual rules and the rule-based prognostic model, we further demonstrate that an individual rule is sufficient to segment population into groups with different risk levels; and the rule-based prognostic model is capable of providing accurate personalized risk assessment by utilizing the relationships between rules and the underlying disease severity. The identified rules also provide solid evidence for designing adaptive monitoring strategies in a treatment population by closely monitoring the patients with warning signals (i.e. endorse increasing risk rules) and less frequent monitoring of the patients with healthy signals (i.e. endorse decreasing risk rules). The advantage of multi-rule based strategy is not large compared with the latest PHQ-9 based strategy because the patients in this studied population has more stable depression severity. Unstable depression progression patterns such as early-high and late-escalating depressed mood have been discovered in clinical practice^9,27^. Therefore, the multi-rule based method has promise to be more effective and universal than the latest PHQ-9 based strategy in a larger population in clinical practice. By integrating the rule discovery, rule evaluation, and rule-based monitoring strategy design into a unified framework, the rule-based method effectively translates the EHR data into actionable knowledge and enhances the efficient use of data-driven evidence in medical decision making.

There are several limitations of our study. First, our depression EHR data provide limited information on patients’ socioeconomic and other clinical factors. As discovered in the literature, factors including race, education level, family structure, psychotic symptoms, and co-morbidities (e.g. anxiety disorder) are likely to be associated with depression progression^27^. Thus, additional data such as providers’ notes or clinical text may lead to the discovery of more risk predictive patterns and improving the accuracy of depression prognostics. Second, due to the lack of knowledge on treatment response scenarios and the lack of adequate depression assessments that cover the whole life span of patients in the depression treatment population, it is challenging to estimate the long-term effectiveness of adaptive monitoring strategies.

In summary, we discovered 12 risk predictive rules from a depression treatment population that can segment individuals into risk subgroups based on their longitudinal patterns. We further developed and evaluated adaptive monitoring strategies based on these identified rules. We established a rule-based analytic framework to automatically leverage the sparse, irregular and time-varying measurements in EHR data to support the monitoring strategy design by integrating the data transformation, rule discovery and rule evaluation. More generally, the proposed method can lead to a better understanding of disease dynamics, more accurate prognostics of disease progressions, and efficient monitoring of a treatment population in clinical practice.

## Electronic supplementary material

Supplementary File
