# Supplementary material for: Data-based Decision Rules to Personalize Depression Follow-up
Source: Sci Rep. 2018 Mar 22;8:5064. doi: 10.1038/s41598-018-23326-1 (PMC5864956; doi:10.1038/s41598-018-23326-1)
Supplement: Supplementary file 1 — Supplementary File [file 41598_2018_23326_MOESM1_ESM.pdf]

Data-based Decision Rules to Personalize Depression Follow-up

Ying Lin, PhD[1,*], Shuai Huang, PhD[2], Gregory E. Simon, MD, MPH[3,4], Shan Liu, PhD[2]

1. Department of Industrial Engineering, University of Houston, 4722 Calhoun Road, Houston, TX 77204, United States
2. Department of Industrial and Systems Engineering, University of Washington, Box 352650, Seattle, WA 98195, United States
3. Kaiser Permanente Washington Health Research Institute, 1730 Minor Ave, Suite 1600, Seattle, WA 98101, United States
4. Psychiatry and Behavioral Sciences, University of Washington, Box 356560, Seattle, WA 98195, United States

[*] Corresponding author: ylin58@uh.edu

eAppendix 1: Additional description on data transformation

Non-random longitudinal patterns

We adopt control theory to further extract some non-random longitudinal patterns in individuals' PHQ-9 scores. Control charts, such as the R chart, S chart and moving range chart, and control rules including the Western Electronic rules (WE) and Nelson (NC) rules are commonly used to capture the non-random patterns in system monitoring[1]. Given the sequential measurements of a dynamic process, a control chart is built to monitor the process over time. It is constructed by a central line for the average value and the upper and lower lines for the control limits which can be determined from historical data. The control rules including the WE rules and NC rules listed in Supplementary eTable 1 can be applied on the control chart to reveal the non-random patterns for the early detection of anomalies in the process. To extend the control rules for capturing non-random patterns in EHR data, we use the moving range control chart which is developed for individual measurements. Denote the measurements on an individual as $[x_1, \ldots, x_T]$, the control chart is constructed by the mean value of observations $\bar{x}$, the average of moving range $\overline{\mathrm{MR}} = \frac{\sum_{i=2}^{T}|x_i - x_{i-1}|}{T-1}$, and the estimated standard deviation of observations $\hat{\sigma} = \overline{MR}/d_2$, where d_2 is a constant that represents the expected value of the moving range of T normal observations when standard deviation equals to 1. Thus the moving range control chart is constructed by

$$\text{Central limit} = \bar{x}$$
$$\text{Upper control limit} = \bar{x} + 3\hat{\sigma}$$
$$\text{Lower control limit} = \bar{x} - 3\hat{\sigma}$$

To detect non-random patterns, control rules listed in Supplementary eTable 1 are applied on the control charts, and a set of binary factors that indicating the observations of non-random patterns on each individual is generated. The control chart on a randomly selected individual is shown in Supplementary eFigure 1, in which the control rules WE1, WE2, WE8, NC1 and NC2 are satisfied.

After eliminating the control rule factors that are supported by less than 1% of the population, we generate 45 risk predictive factors in total, and provide the summarization of them in Supplementary eTable 3.

Supplementary eTable 1: The Western Electric and Nelson control rules

Control Rules	Content
**Western Electronic Rule 1 (WE1)**	One point falls above the upper 3σ limit
**Western Electronic Rule 2 (WE2)**	Two out of three consecutive points fall above the upper 2σ limit
**Western Electronic Rule 3 (WE3)**	Four out of five consecutive points fall above the upper 1σ limit
**Western Electronic Rule 4 (WE4)**	Eight consecutive points fall above centerline
**Western Electronic Rule 5 (WE5)**	One point falls below the lower 3σ limit
**Western Electronic Rule 6 (WE6)**	Two out of three consecutive points fall below the lower 2σ limit
**Western Electronic Rule 7 (WE7)**	Four out of five consecutive points fall below the lower 1σ limit
**Western Electronic Rule 8 (WE8)**	Eight consecutive points fall below centerline

**Western Electronic Rule 9 (WE9)**	Fifteen consecutive points fall between lower and upper 1σ limits
**Western Electronic Rule 10 (WE10)**	Eight consecutive points fall beyond the 1σ limits
**Nelson Rule 1 (NC1)**	Night consecutive points fall on the same side of centerline
**Nelson Rule 2 (NC2)**	Six consecutive increasing or decreasing points
**Nelson Rule 3 (NC3)**	Fourteen consecutive points alternate up and down

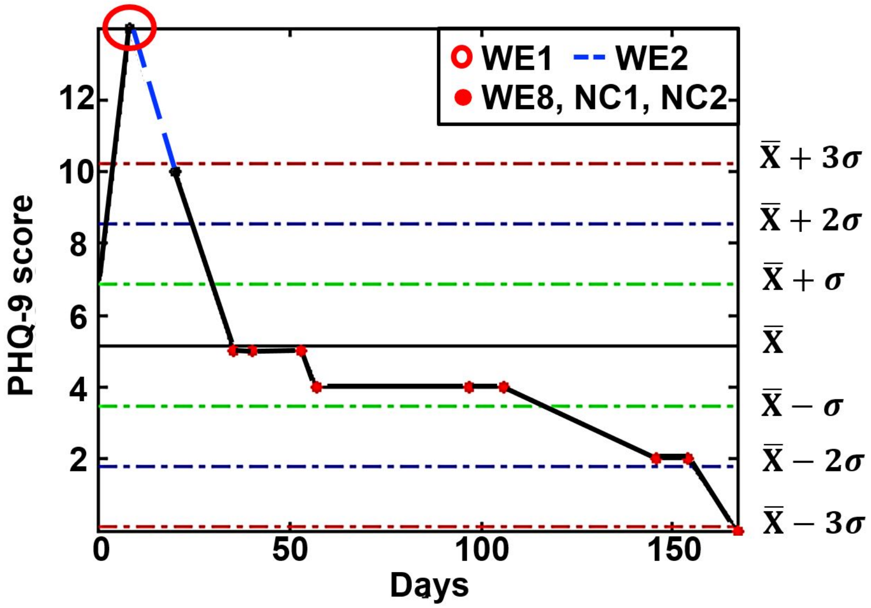

Supplementary eFigure 1. The moving range control chart on individual measurements. The measurements satisfy control rules: WE1, WE2, WE8, NC1, and NC2.

eAppendix 2: Additional description on rule discovery and evaluation

RuleFit model description

RuleFit identifies the candidate rules by "rule generation" and "rule pruning" phases[2]. "Rule generation" phase estimates a number of trees with each tree being estimated on a relatively homogeneous subpopulation generated by bootstrapping the original dataset, and regards each path from root to leaf node as a rule. The height of each decision tree controls the complexity of rules (i.e. the number of risk factors included in each rule), which is bounded from above by 3 in our study. "Rule pruning" phase considers each rule as a "variable" with each element indicates the endorsement of each patient and applies a sparse regression model to select the minimum set of rules that are predictive to the outcomes.

Item response theory (IRT)

IRT is often used in psychometric problems to infer students' abilities, attitudes, or personalities by gathering evidence from questionnaire responses or tests[3]. It assigns each individual a latent variable θ denoting the underlying disease severity, and models the likelihood of endorsement of each rule as a function of both the individual's disease severity and the association between this rule and the disease severity, which corresponds to information on where the rule stands in the

disease severity continuum and how predictive the rule is. Denote the probability of an endorsement of rule R_l given the disease severity θ as $P_l(R_l = 1|\theta)$, and the probability of un-endorsement as $P_l(R_l = 0|\theta)$, the probability is modeled by a monotonically increasing function called the item characteristic curve (ICC) in the following form:

$$\log\frac{P_l(R_l=1|\theta)}{P_l(R_l=0|\theta)} = a_l(\theta - b_l), \qquad (1)$$

where b_l is the item difficulty parameter that represents the disease severity required to achieve a 50% chance of endorsement of rule R_l, i.e. $P_l(R_l = 1|\theta = b_l) = P_l(R_l = 0|\theta = b_l) = 0.5$. a_l is the item discrimination parameter for R_l that determines the amount of change in the log odds, $P_l(R_l = 1|\theta)/P_l(R_l = 0|\theta)$, for one unit change in the disease severity. A larger b_l indicates the individuals endorsing rule R_l is more likely to have higher disease severity and a larger a_l means that rule R_l is more sensitive to the small changes in the disease severity. The parameters in item characteristic curves, $\{a_l, b_l, \theta\}$, can be estimated by the Markov Chain Monte Carlo (MCMC) algorithm[4]. We use the ICCs to associate the rule endorsements with underlying disease severity.

eAppendix 3: Additional results

Supplementary eTable 2: Significant predictors identified by logistic regression model ($\alpha = 0.05$). The logistic regression model uses all factors generated from predicting period to predict the probability of progressing to low-risk group in responding period.

Risk factors	Coefficient	SE	P-value
**Intercept**	4.49	1.88	0.02*
**Age between 30 to 44 years old**	0.19	0.24	0.43
**Age between 45 to 64 years old**	-0.04	0.23	0.86
**Age greater or equal to 65 years old**	0.53	0.27	0.05*
**Sex (1 = female)**	-0.40	0.16	0.01*
**Number of observations**	0.04	0.06	0.49
**Time stamp of first observation**	0.00	0.00	0.81
**Observing density**	0.21	1.61	0.90
**First Charlson comorbidity score**	0.01	0.33	0.98
**Maximal Charlson comorbidity score**	0.18	0.70	0.80
**Minimal Charlson comorbidity score**	0.18	0.71	0.80
**Range of Charlson comorbidity score**	0.07	1.11	0.95
**Average Charlson comorbidity score**	0.08	0.59	0.89
**Median of Charlson comorbidity score**	-0.07	0.45	0.88
**25% percentile of Charlson comorbidity score**	-0.48	0.66	0.47
**75% percentile of Charlson comorbidity score**	0.01	0.59	0.99
**Volatility of Charlson comorbidity score**	-0.19	2.08	0.93
**First item 9’s score**	0.02	0.14	0.92
**Maximal item 9’s score**	0.17	0.68	0.81
**Minimal item 9’s score**	-0.11	0.80	0.89
**Range of item 9’s score**	0.23	0.69	0.75
**Average item 9’s score**	0.16	1.31	0.91
**Median of item 9’s score**	0.03	0.41	0.94
**25% percentile of item 9’s score**	0.19	0.57	0.74
**75% percentile of item 9’s score**	-0.20	0.45	0.65
**Volatility of item 9’s score**	-0.45	0.96	0.64
**First PHQ-9 score**	0.03	0.03	0.30
**Latest PHQ-9 score**	-0.06	0.03	0.02*
**Maximal PHQ-9 score**	-0.06	0.11	0.59
**Minimal PHQ-9 score**	-0.08	0.11	0.51
**Range of PHQ-9 score**	0.00	0.11	0.99
**Average PHQ-9 score**	-0.10	0.18	0.58
**Median of PHQ-9 score**	0.04	0.06	0.54
**25% percentile of PHQ-9 score**	-0.04	0.09	0.66
**75% percentile of PHQ-9 score**	-0.09	0.08	0.26
**Volatility of PHQ-9 score**	0.04	0.18	0.84
**Deepest increase between consecutive PHQ9 scores**	-0.01	0.04	0.85
**Deepest decrease between consecutive PHQ9 scores**	-0.01	0.04	0.85
**Volatility of difference between nearby PHQ9 scores**	-0.02	0.05	0.75

**Percentage of depression free**	-0.95	1.77	0.59
**Percentage of mild depression**	-0.56	1.56	0.72
**Percentage of moderate depression**	-0.61	1.45	0.68
**Percentage of moderately severe depression**	0.06	1.44	0.97
**Percentage of severe depression**	0.68	1.53	0.66

Supplementary eTable 3: Means and standard deviations (in brackets) of 45 risk factors (N=1,762 patients).

**Risk factors**	**Low-risk group (Y_i=1), 876(49.72%)**	**Depressive group (Y_i=0), 886(50.28%)**
Age n(%)		
**≤ 30 years old**	102(11.64%)	124(14.00%)
**30 to 44 years old**	234(26.71%)	240(27.09%)
**45 to 64 years old**	358(40.87%)	416(46.95%)
**≥ 65 years old**	182(20.78%)	106(11.96%)
Sex n(%)		
**Female**	578(65.98%)	643(72.57%)
**Male**	298(34.02%)	243(27.43%)
**Statistical Summarization**		
Charlson comorbidity score		
**First observation**	0.66(1.21)	0.65(1.24)
**Median observation**	0.67(1.24)	0.68(1.27)
**Maximal observation**	0.71(1.28)	0.73(1.30)
**Minimal observation**	0.60(1.18)	0.62(1.21)
**Range of observations**	0.10(0.46)	0.11(0.47)
**Mean of observations**	0.67(1.23)	0.68(1.25)
**Volatility of observations**	0.06(0.25)	0.06(0.26)
**25% percentile of observations**	0.62(1.18)	0.64(1.22)
**75% percentile of observations**	0.69(1.26)	0.71(1.28)
9th question score		
**First observation**	0.40(0.79)	0.73(1.00)
**Median observation**	0.24(0.54)	0.62(0.86)
**Maximal observation**	0.70(1.10)	1.27(1.17)
**Minimal observation**	0.06(0.27)	0.24(0.56)
**Range of observations**	0.64(0.93)	1.04(1.03)
**Mean of observations**	0.31(0.51)	0.69(0.77)
**Volatility of observations**	0.31(0.43)	0.50(0.49)
**25% percentile of observations**	0.11(0.34)	0.36(0.64)
**75% percentile of observations**	0.49(0.77)	1.02(1.02)
PHQ-9 score		
**First observation**	11.69(6.57)	16.31(6.05)
**Median observation**	9.45(5.31)	15.47(5.18)
**Maximal observation**	11.42(6.23)	19.88(4.76)
**Minimal observation**	5.11(4.06)	10.71(5.60)
**Range of observations**	9.31(5.60)	9.17(5.26)
**Mean of observations**	9.60(4.66)	15.38(4.63)

**Volatility of observations**	4.20(2.49)	4.18(2.36)
**25% percentile of observations**	6.59(4.37)	12.39(5.29)
**75% percentile of observations**	12.57(5.71)	18.37(4.65)
**Percentage of healthy states**	0.23(0.29)	0.05(0.13)
**Percentage of mildly depressive states**	0.32(0.28)	0.14(0.21)
**Percentage of moderately depressive states**	0.23(0.24)	0.24(0.26)
**Percentage of moderately severe states**	0.14(0.20)	0.27(0.26)
**Percentage of severely depressive states**	0.09(0.18)	0.29(0.32)
**Progression Trajectories**		
**Number of observations**	4.47(2.11)	4.34(2.10)
**Time of first observation since initial treatment (days)**	217.64(413.16)	239.17(418.29)
**Observing density**	0.04(0.03)	0.04(0.05)
**Latest PHQ-9 score**	7.53(5.41)	14.14(6.35)
**Deepest increasing between consecutive PHQ-9 scores**	4.07(4.25)	4.79(4.55)
**Deepest decreasing between consecutive PHQ-9 scores**	6.72(4.88)	6.16(4.59)
**Volatility of difference between nearby PHQ-9 score**	5.52(4.26)	5.63(4.01)
**Non-random Longitudinal Pattern**		
WE1 n(%)		
**Support**	253(28.88%)	191(10.84%)
**Not support**	623(71.12%)	695(89.16%)
WE2 n(%)		
**Support**	112(12.79%)	88(4.99%)
**Not support**	764(87.21%)	798(95.01%)
WE5 n(%)		
**Support**	209(23.86%)	219(12.43%)
**Not support**	667(76.14%)	667(87.57%)
WE6 n(%)		
**Support**	131(14.95%)	97(5.51%)
**Not support**	745(85.05%)	789(94.49%)

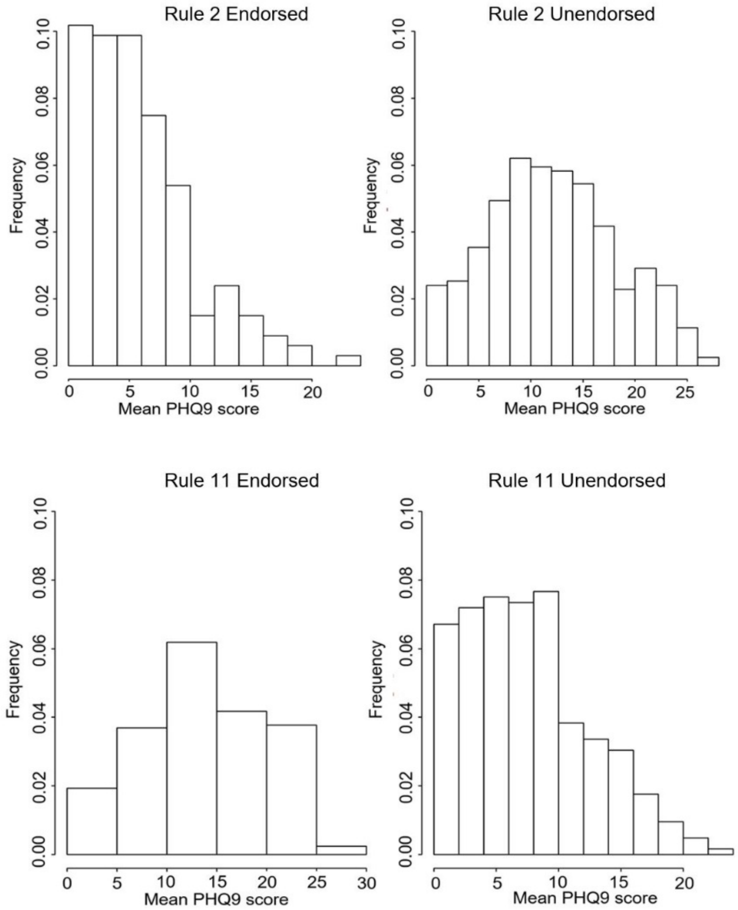

Supplementary eFigure 2. Distribution of average PHQ-9 scores in rule endorsing and un-endorsing groups. Plot on randomly selected decreasing risk rule 2 (top) and increasing risk rule 11 (bottom).

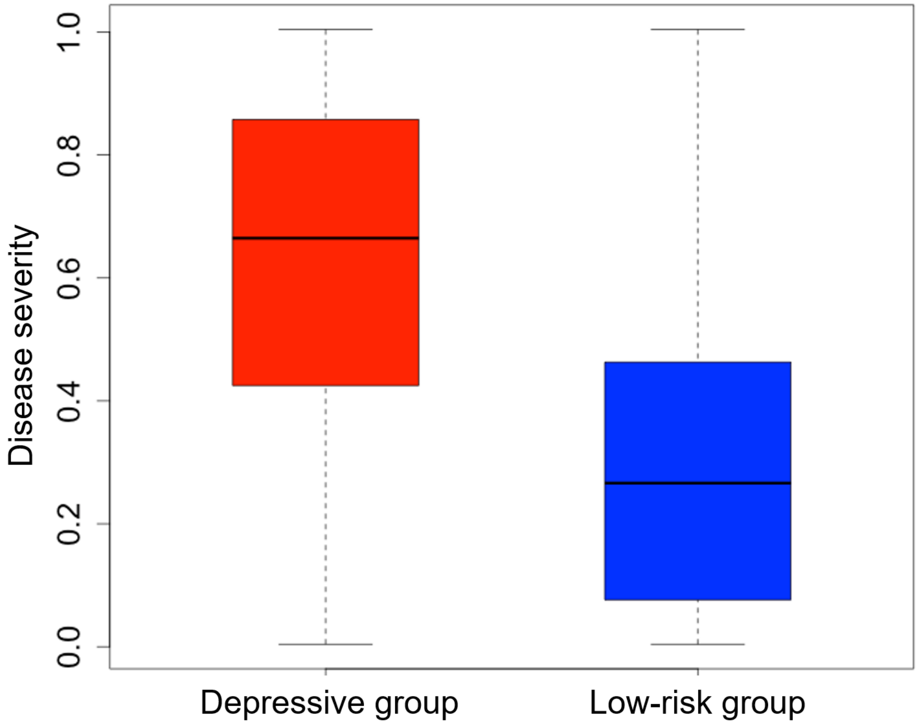

Supplementary eFigure 3. The boxplots of disease severities in depressive group (average PHQ-9 score ≥ 10) and low-risk group (average PHQ-9 score <10)

Reference:

1. Montgomery, D.C., Design and analysis of experiments. John Wiley & Sons (2008).
2. Friedman, J. & Popescu, B.E. Predictive Learning via rule ensemble. Annals of Applied Statistics. 2 (3), 916–954 (2008).
3. Lord, F.M. Applications of item response theory to practical testing problems. *Routledge* (1980).
4. Baker, F.B. Item response theory: Parameter estimation techniques. *CRC Press* (2004).
